# Role of Autofluorescence in Inflammatory/Infective Diseases of the Retina and Choroid

**DOI:** 10.1155/2014/418193

**Published:** 2014-04-01

**Authors:** Ahmed Samy, Sue Lightman, Filis Ismetova, Lazha Talat, Oren Tomkins-Netzer

**Affiliations:** ^1^Moorfields Eye Hospital, City Road, London EC1V 2PD, UK; ^2^UCL Institute of Ophthalmology, London EC1V 9EL, UK

## Abstract

Fundus autofluorescence (FAF) has recently emerged as a novel noninvasive imaging technique that uses the fluorescent properties of innate fluorophores accumulated in the retinal pigment epithelium (RPE) to assess the health and viability of the RPE/photoreceptor complex. Recent case reports suggest FAF as a promising tool for monitoring eyes with posterior uveitis helping to predict final visual outcome. In this paper we review the published literature on FAF in these disorders, specifically patterns in infectious and noninfectious uveitis, and illustrate some of these with short case histories.

## 1. Introduction

Fundus autofluorescence (FAF) is a noninvasive imaging modality that provides a topographical retinal map of lipofuscin that has accumulated in the retinal pigment epithelium [1]. FAF, first viewed as pseudofluorescence during florescence angiography predye administration [2], has only recently been recognized as a useful indicator for disease activity and extent of retinal pigment epithelium (RPE) damage, assisting an in-depth understanding of the pathophysiologic mechanisms in a wide variety of retinal diseases. As such, it is attracting the attention of many uveitis specialists to investigate its usefulness in various uveitic diseases.

In healthy human retina, the photoreceptor outer segments are shed daily, phagocytosed, and digested by the RPE [3]. Lipofuscin, the dominant fluorophore in the retina, is believed to be the result of accumulation of incompletely degraded products of photoreceptor outer segments in the RPE cytosol [4–6]. Lipofuscin inhibits lysosomal degradation, is photoreactive, and produces oxygen radicals that can lead to a reduced phagocytic capacity of the RPE and eventually RPE cell death and photoreceptor loss [7–12]. Lipofuscins constitute a complex mixture of bisretinoids and contain a broad range of fluorophores with an excitation spectrum ranging from 300 to 600 nm and an emission spectrum from 480 to 800 nm [13]. Retinal photoreceptor degeneration, secondary to retinal disease, can cause visual loss in patients with uveitis. Retinal damage in retinal antigen-induced experimental autoimmune uveitis (EAU), an animal model that resembles some types of human uveitis, has been attributed to blood-borne activated macrophages, which are known to generate various toxic agents [14, 15]. Macrophages and T cells typically infiltrate the retina in the early stages of EAU (days 11-12 after immunization). However, in day 5 after immunization, studies have shown peroxynitrite-mediated nitration of photoreceptor mitochondrial proteins [16], leading to mitochondria dysregulation and cell death [17]. Lipofuscins are thought to represent the breakdown product of various retinal proteins as a result of oxidative damage which is thought to play a role in uveitic diseases [18]. Visualization of lipofuscin accumulation in the RPE reflects disease activity and, in a clinical setting, the intensity of FAF correlates with the amount and distribution of lipofuscin in the RPE layer, serving as a measure of RPE health and function [19]. Therefore, accumulation of lipofuscin in the RPE indicates that oxidative cellular damage has occurred or is occurring [20]. An increase in FAF (hyperautofluorescence) is expected in the presence of increased metabolic activity of the RPE, a predictor of dysfunction, and a decrease in FAF (hypoautofluorescence) with the loss of photoreceptors or the RPE [1].

**Figure 1:**
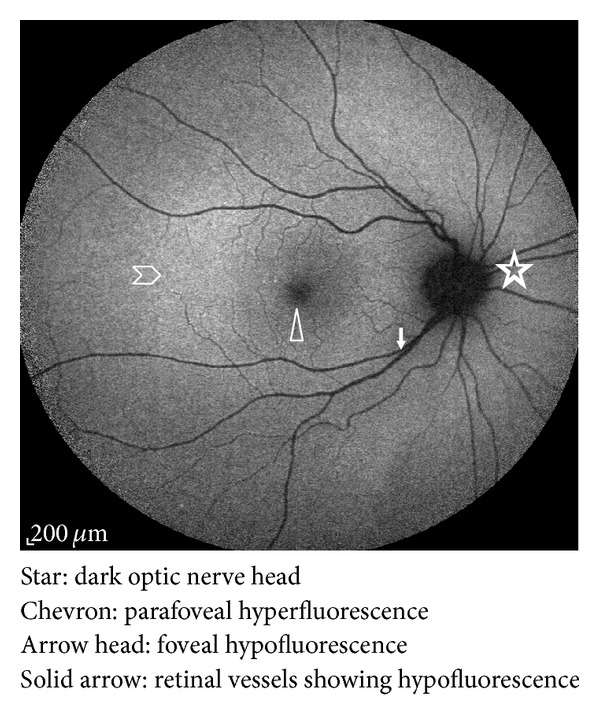
Autofluorescence distribution in a normal eye fundus. It is the highest in the posterior pole and gradually diminishes toward the periphery; it also shows hypoautofluorescence over the fovea, the optic nerve head, and retinal vessels.

Autofluorescence imaging in normal eyes shows a dark optic nerve head because of the absence of RPE and lipofuscin. Retinal vessels would also appear dark as they block FAF that would otherwise originate from the underlying RPE [13]. The fovea is hypoautofluorescent because of absorption of light by the luteal pigment [21]. The parafoveal region is slightly hyperautofluorescent due to increased RPE and photoreceptor metabolic activity (Figure 1).

Alterations in FAF have been described in several posterior uveitic syndromes and can help to distinguish between them, provide information on the detection and localization of inflammatory disease activity, and can potentially serve as a prognostic marker for visual outcome. Different autofluorescence patterns are reported in infectious and noninfectious uveitides as well as masquerade syndromes [22]. Most reports share the common finding of hyperautofluorescence with increased disease activity that fades and darkens as the inflammation subsides [23]. In this review we examine FAF patterns in infectious and noninfectious posterior uveitis and discuss the change in these patterns in relation to disease activity.

## 2. Noninfectious Uveitis

### 2.1. Multifocal Choroiditis (MFC) and Punctate Inner Choroidopathy (PIC)

Punctate inner choroidopathy (PIC) is an uncommon recurrent idiopathic inflammatory disease affecting young myopic women [24], and while both eyes are usually involved this may not occur simultaneously. Clinically, PIC lesions are multiple small yellow-white spots (100–200 *μ*m) with fuzzy borders at the level of the inner choroid and retina. Multifocal choroiditis (MFC) is usually a bilateral condition, which appears as multiple choroidal inflammatory lesions involving the posterior pole and peripheral retina, which may be accompanied by anterior chamber inflammation and vitritis [25]. Symptoms of both conditions usually include photopsias and decreased visual acuity. Choroidal neovascular (CNV) membranes develop in both conditions in up to 76.9% of patients, usually within a year of presentation [26].

**Figure 2:**
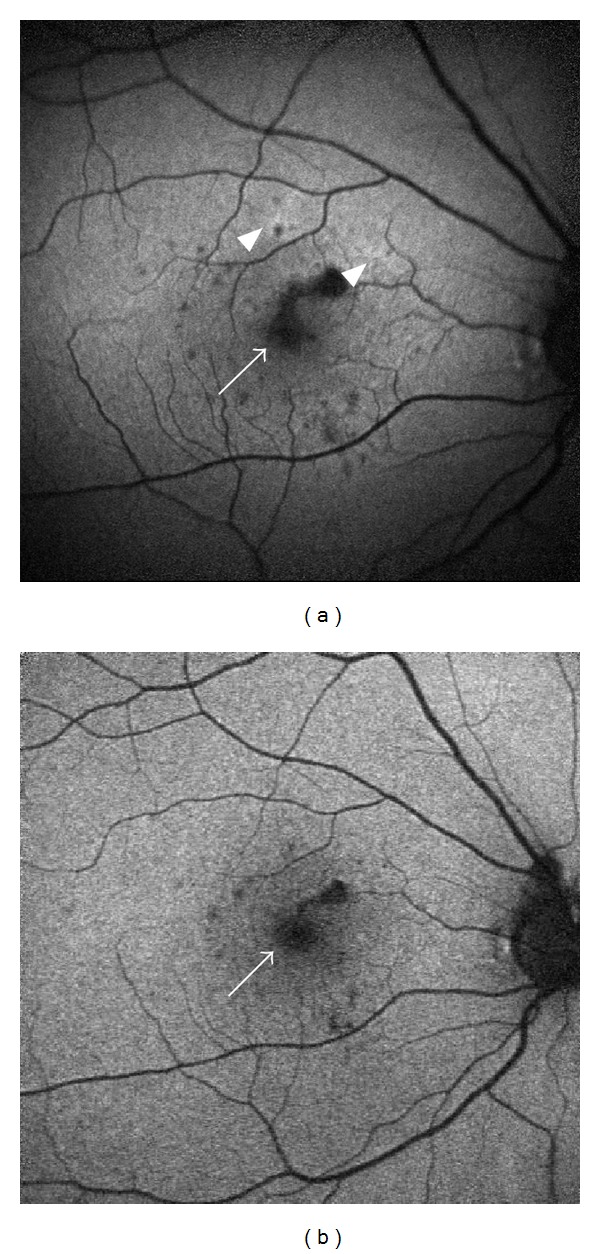
Fundus autofluorescence (FAF) images of a young female with punctate inner choroiditis. (a) FAF shows hyperautofluorescence halos (arrow heads) and multiple hypoautofluorescent spots (arrow). The spots are surrounded by a hyperautofluorescent halo, denoting continued cellular damage and ongoing active inflammation. (b) FAF captured 5 months after immunosuppression was started shows diminished hyperautofluorescence and less hypoautofluorescent spots.

MFC and PIC have a pronounced effect on the morphology and function of the RPE [27]. A recent study conducted on 36 eyes with MFC demonstrated that the number of hypoautofluorescent spots on FAF is far greater than the chorioretinal scars seen on clinical examination. They classified these hypoautofluorescent spots into two patterns according to size [28]. They reported that spots >125 *μ*m were related to visible scars and that those hypoautofluorescent spots <125 *μ*m in diameter were not clinically visible. The smaller spots appeared to cluster around areas of CNV and in some cases appeared to precede the clinically apparent choroidal lesions. The spots seen on FAF are likely to reflect a more accurate measure of disease activity and cellular damage than clinical examination alone, and FAF is less invasive than fluorescein angiography (FA) or indocyanine green angiography (ICG) [29]. A retrospective study of 8 patients with PIC used FAF imaging to assess response of active PIC lesions to immune-modulatory treatment (IMT). Hyperautofluorescence was seen surrounding active PIC lesions and associated CNVs. Hypoautofluorescence occurred when the lesions responded to treatment (Figure 2) and persistence of hyperautofluorescence was associated with a risk of recurrence or continuing active disease. The authors hypothesized that, in the inactive phase of the disease, RPE death results in areas of hypoautofluorescence, although in some instances hypofluorescence at the edges of active lesions may be caused by cellular swelling which could be misleading [30].

### 2.2. Birdshot Chorioretinopathy

**Figure 3:**
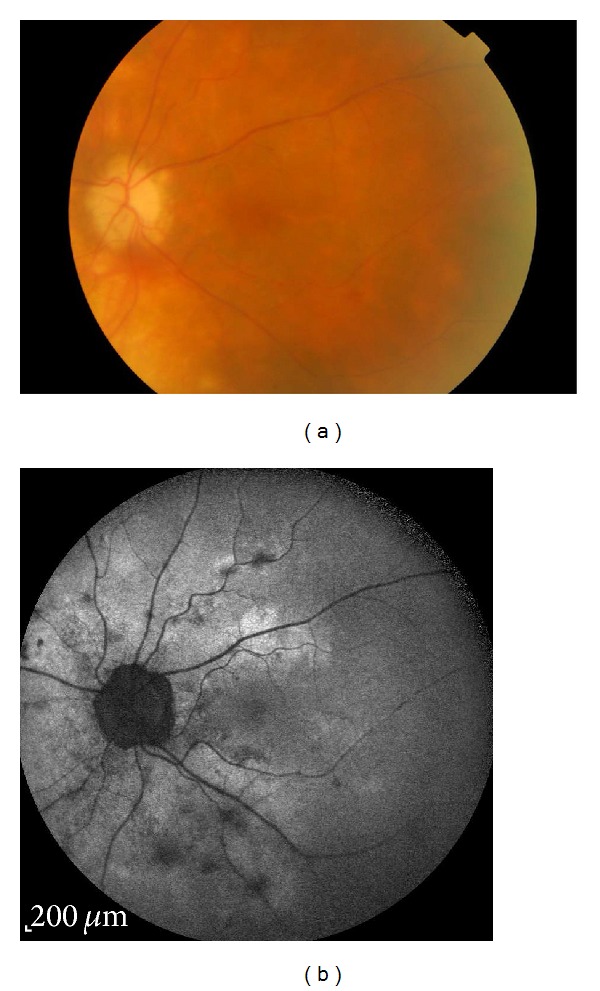
Areas of hypoautofluorescence in birdshot chorioretinopathy. (a) corresponds to the typical lesions in most parts and (b) shows more widespread lesions than the ophthalmologically visible area of involvement.

Birdshot chorioretinopathy (BSCR) is a chronic, bilateral posterior uveitis characterized by hypopigmented deep yellow lesions scattered throughout the posterior pole [25, 31, 32]. The disease is more common in middle-aged Caucasians and has a strong correlation with the HLA-A29 antigen [33, 34]. There is widespread consensus that the choroid is the initial site of inflammation due to T-cell accumulation resulting in the distinct BSCR lesions, with a secondary effect on the RPE and photoreceptor layers [35, 36]. Active disease usually presents with mild vitritis, vasculitis, optic disc swelling, and cystoids macular oedema (CME). FAF studies on BSCR patients showed discrete areas of hypoautofluorescence, which did not always correspond to clinically visible birdshot lesions (Figure 3) or were larger and more diffused than any visible lesions [22, 37]. A 17% incidence of linear perivascular hypoautofluorescence that correlates with clinical findings has also been reported [38]. These studies identified that about 80% of eyes with BSCR had more numerous and more easily recognized abnormalities on FAF than on fundus photography, with similar findings documented with the more invasive ICG angiography [38].

Hypoautofluorescent lesions were better correlated with visible BSCR lesions in eyes with advanced disease [22]. Patients with predominantly choroidal inflammation without overlying RPE damage have fewer FAF findings, with prolonged choroidal inflammation resulting in eventual RPE damage and subsequent photoreceptor loss, related to vision and visual field changes in these patients. Patients with chorioretinitis, including a BSCR patient, demonstrated that visual field changes correlated with areas of reduced FAF in both eyes [39]. In BSCR patients with placoid areas of hypofluorescence in the macula, there was a poorer visual outcome and thinner macula on optical coherence tomography (OCT) than patients with no macular involvement [38]. These observations serve to support the argument of initiating immunosuppressive therapy in patients with BSCR lesions before the onset of RPE damage, as evidenced by the appearance of overlying hypoautofluorescent areas [31].

### 2.3. Multiple Evanescent White Dot Syndrome

Multiple evanescent white dot syndrome (MEWDS) is a retinochoroiditis that is typically described in young myopic females and is occasionally preceded by a viral prodrome. Classically, patients complain of sudden visual loss in the form of central or paracentral scotomas or enlarged blind spot. Fundus examination typically reveals multiple small yellow-white spots in the posterior pole of various sizes ranging from 100 *μ*m to 200 *μ*m, as well as fine orange granularities or specks at the fovea [40–42]. OCT performed on the affected areas during the acute phase reveals hyperreflective lesions in the subretinal space and multifocal attenuation and disruption of the photoreceptor inner/outer segment (IS/OS) junction [43, 44]. In one study a strong correlation was noted between hypofluorescent pots on ICG and disruption of the IS/OS junction on OCT, supporting the hypothesis that the disease initially starts in the photoreceptor layer and not in the choroid [43]. The disease has a favourable prognosis and is usually self-limiting, with full recovery of vision within weeks to months.

**Figure 4:**
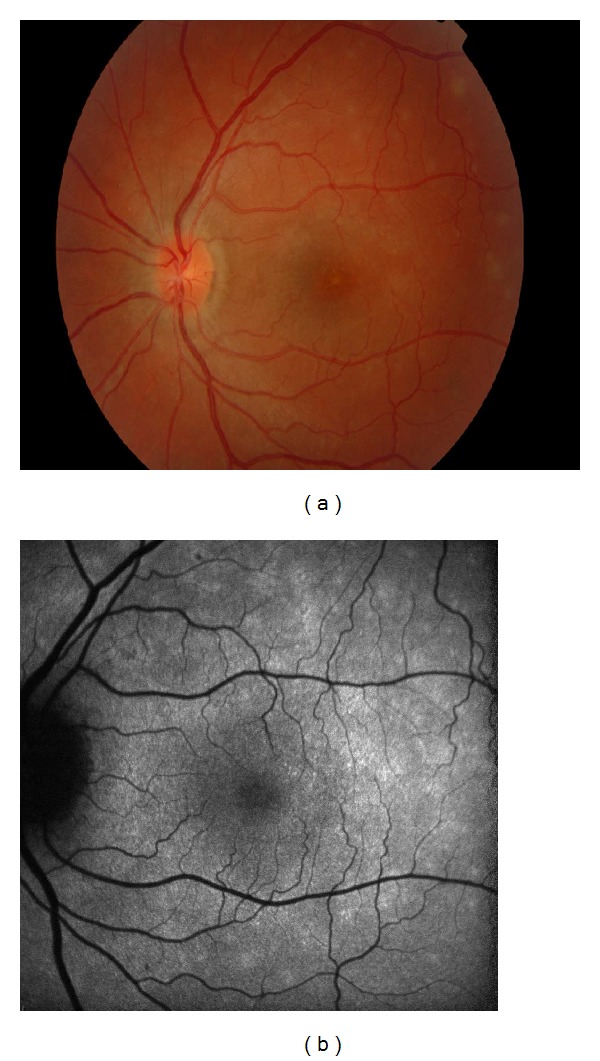
Areas of hyperautofluorescence in multiple evanescent white dot syndrome. (a) Colour fundus images showing multiple small yellow-white spots and fine orange granularities at the fovea. (b) FAF of the same eye showing multiple ill-defined spots of hyperautofluorescence.

During the acute phase of the disease, FAF demonstrates multiple ill-defined spots of hyperautofluorescence in correspondence with the clinically visible white spots (Figure 4), which also correspond to the lesions seen on FA. This hyperautofluorescence pattern may be secondary to disrupted or misaligned photoreceptors or to an increased rate of shedding of the photoreceptor outer segments that are related to active inflammation. Following resolution of the inflammation, the hyperautofluorescent lesions disappear [45].

### 2.4. Acute Posterior Multifocal Placoid Pigment Epitheliopathy (APMPPE)

Acute posterior multifocal placoid pigment epitheliopathy (APMPPE) is an idiopathic bilateral condition typically affecting healthy young adults and characterized by rapid loss of central vision with multiple round, placoid, and gray-white lesions at the level of the RPE [46]. It presents with a distinct FAF pattern of hypoautofluorescence during the acute phase, related to a masking effect secondary to overlying oedematous retinal cells. As the lesions and oedema resolve, a hyperautofluorescence pattern emerges due to photoreceptor loss and release of lipofuscin and other fluorophores [47].

### 2.5. Primary Intraocular Lymphoma (Primary Vitreoretinal Lymphoma)

**Figure 5:**
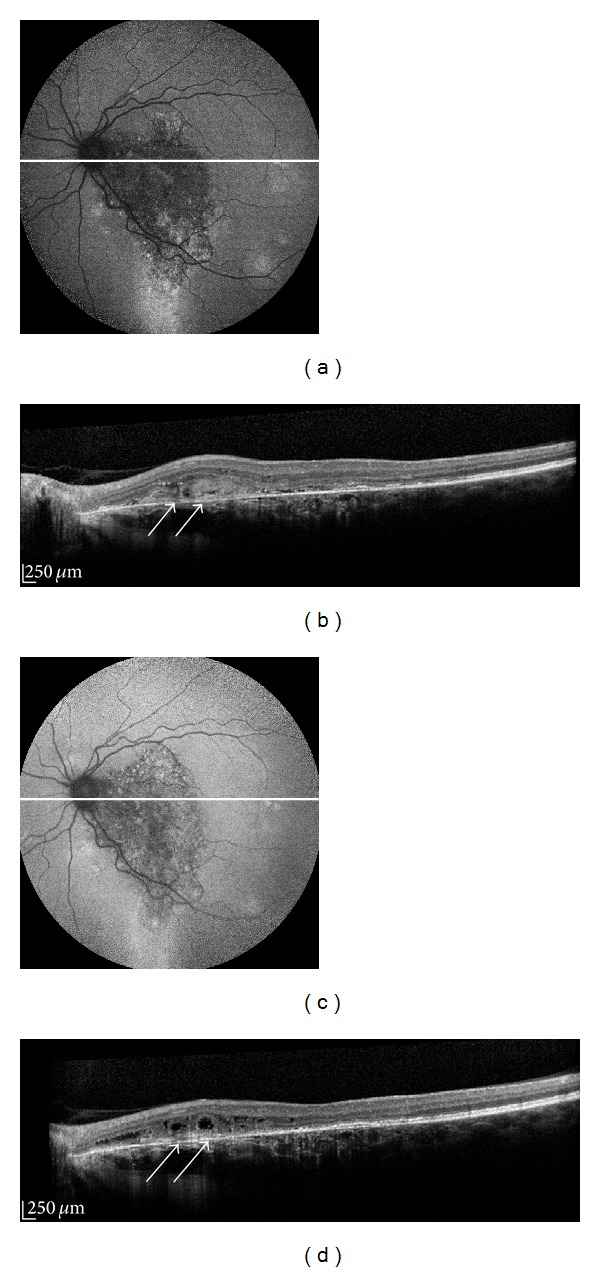
Fundus autofluorescence (FAF) images of a male with primary vitreoretinal lymphoma (PVRL). (a) FAF images of a patient with PVRL showing predominantly hyperautofluorescence in the form of granular hyper- and hypoautofluorescence, (b) OCT scan showing areas of nodular hyperreflective spots at the level of the RPE (arrows). (c) 2 years following the treatment the FAF image shows less marked granular hyperautofluorescence, as well as a fading of the nodular RPE hyperreflective spots previously noted on OCT ((d), arrows).

Primary ocular non-Hodgkin's lymphoma, commonly referred to as primary intraocular lymphoma (PIOL) [48] or recently suggested to be renamed primary vitreoretinal lymphoma (PVRL), is a subset of primary central nervous system lymphoma (PCNSL). It is an aggressive neoplasm, most frequently of B-lymphoid cell origin and rarely of T-lymphoid cell origin [49]. It may take up to 24 months for the diagnosis of PVRL to be established with a median survival period of 31 months [50–52]. Eighty percent of patients with PVRL will eventually develop CNS lymphoma while 20% of PCNSL cases will develop ocular involvement [53]. Typically, patients present in the 5th to 7th decade with a masquerade syndrome of a chronic intermediate uveitis [54, 55]. Imaging of the eye and brain is the first step in evaluating these patients. However, patterns of FAF in eyes with PVRL may be variable and confusing. Several studies compared the sensitivity and predictive values of FA, spectral-domain OCT (SD-OCT), and FAF images in eyes with known PVRL [56, 57]. They found that a granular autofluorescence pattern could be seen in majority of eyes with active disease. Furthermore, this granular FAF pattern was also observed in some eyes where the classic leopard spot pattern on FA was not clear or when FA could not be performed. Hyperautofluorescent spots appeared to correlate with the hypofluorescent spots on FA and the nodular hyperreflective spots on OCT (Figure 5), all of which were suggestive of active disease. Hyperautofluorescence on FAF is thought to indicate RPE involvement by the lymphomatous infiltrates in the sub-RPE space. It is also possible that the hyperautofluorescence pattern seen is the result of lipofuscin accumulation in the RPE cells adjacent to the tumour [56]. Hypoautofluorescence areas may be caused by blockage of autofluorescence by the infiltrating tumour cells or RPE atrophy which can result from tumour resolution [57]. Hence, abnormal autofluorescence can be helpful in raising the possibility of lymphoma or recurrence in a patient with known PVRL. Since PVRL is a potentially fatal malignancy, early and accurate diagnosis is crucial.

### 2.6. Vogt-Koyanagi-Harada (VKH) Disease

Vogt-Koyanagi-Harada (VKH) disease is a bilateral granulomatous panuveitis associated with an autoimmune reaction against melanocytes and associated with multisystemic involvement [58, 59]. During the acute phase of the disease patients can present with bilateral panuveitis and exudative retinal detachments [58, 60, 61]. It is believed that these detachments and the pinpoint leakage on FA are the result of granulomas in the choroid causing alterations in the RPE and patients should be treated promptly to prevent permanent ocular damage and visual loss. Some patients continue to progress and develop chronic disease with choroidal depigmentation and RPE clumping, resulting in a sunset glow fundus [58, 60]. Koizumi et al. examined the FAF images of 10 eyes from five patients with acute VKH. These patients were followed for up to 6 months and analyzed retrospectively [62]. They classified FAF findings into two distinct patterns; the first was described in acute patients who received early intensive immunosuppression and showed mild hyperautofluorescence, which diminished in size and intensity during followup and returned to normal upon disease remission. The second pattern was seen in patients who either were not treated or in whom treatment was delayed. These showed scattered and widespread areas of hyperautofluorescence, which corresponded to areas of ICG hypofluorescence. This pattern resolved within 6 months to leave an intermingled pattern of hyper- and hypoautofluorescent spots throughout the retina. In a separate case report, a target-like pattern of hyper- and hypoautofluorescence areas was noted, reflecting changes attributed to the presence of serous retinal detachment [63]. During the chronic phase of the disease FAF is generally normal as sunset glow fundus is not related to RPE loss, but rather to postinflammatory depigmentation or loss of choroidal melanocytes [64, 65]. Thus FAF may assist in identifying the acute phase of VKH and disease remission.

## 3. Infectious Uveitis

### 3.1. Serpiginous-Like Choroiditis (SLC)

**Figure 6:**
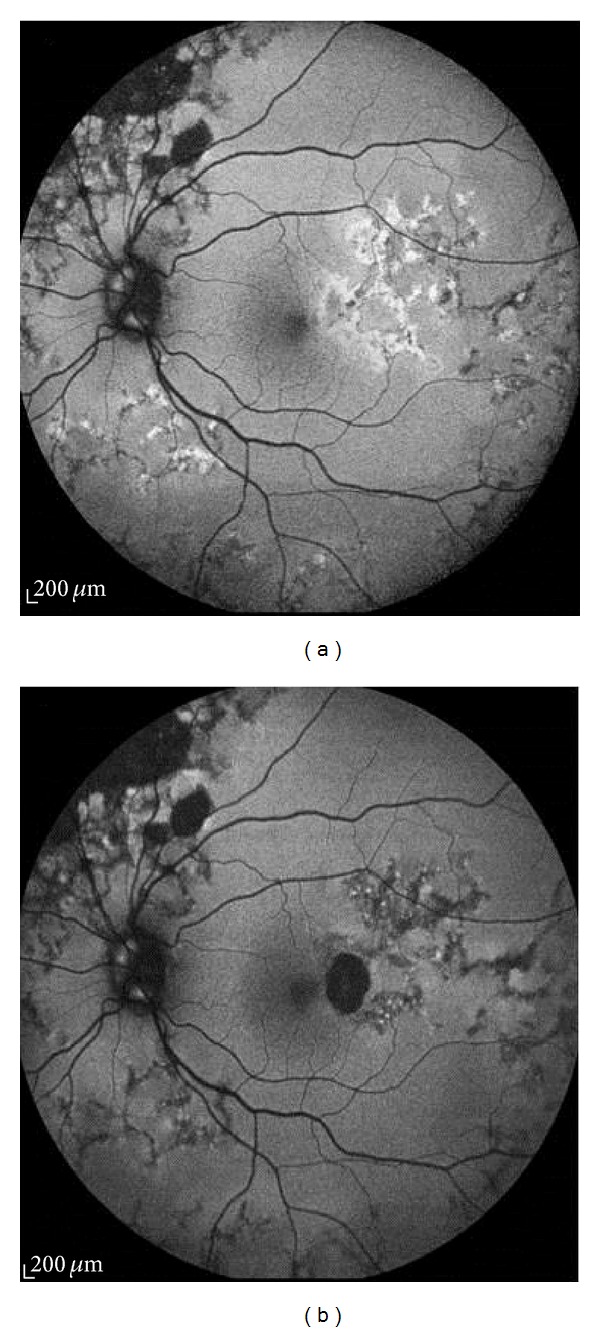
Fundus autofluorescence image of left eye of a male patient with tuberculous choroiditis. (a) An ill-defined halo of hyperautofluorescence corresponding to the active lesion, giving it a diffuse, amorphous appearance. (b) Two months later, a thin rim of hypoautofluorescence appears surrounding the predominantly hyperautofluorescent lesion.

Serpiginous choroiditis is a chronic, progressive, recurrent inflammatory disease affecting primarily the inner choroid and RPE [66]. Conversely, serpiginous-like choroiditis of presumed tubercular etiology (SLC) [67, 68] is a distinct clinical entity that begins with multifocal choroidal lesions that coalesce and progress in a serpiginoid pattern at the posterior pole of the eye [68, 69]. SLC manifests as multifocal placoid lesions that advance in a serpiginoid fashion and become confluent. The diagnosis is supported by a positive interferon-*γ* release assay or PPD skin test, absence of other known causes of infectious and noninfectious uveitis, and a response to antituberculosis therapy [70, 71]. The choriocapillaris has been shown to be the most affected layer in serpiginous choroiditis (SC) and most likely in SLC [72]. In a prospective study on four eyes in 3 patients with SLC changes in high-resolution SD-OCT scans were compared with FAF scans [73]. During the acute stage, there was an ill-defined area of hyperautofluorescence around the lesion. The SD-OCT passing through this area showed a localized, indistinct area of hyperreflectivity in the outer retinal layers involving the RPE and there was no increased backscatter from the inner choroid. As the lesions began resolving, they became well defined and acquired a thin border of hypoautofluorescence though remaining predominantly hyperautofluorescent centrally. The SD-OCT scan through the hyperautofluorescent area showed disappearance of the hyperreflectivity in distinct areas that were replaced by irregular, hyperreflective lumpy elevations of the outer retinal layers. At this stage, there was increased reflectance from the choroidal layers due to the attenuating RPE-photoreceptor complex. As the lesions healed further, they appeared stippled with predominant hypoautofluorescence. The SD-OCT scan showed loss of RPE, IS/OS junction, while the increased reflectance from the choroid persisted. In this study all eyes with active lesions of SLC illustrated progressive changes in the outer retinal layers on OCT scans that correlated with the FAF changes. The FAF images demonstrated the transition from initial hyperautofluorescence seen in the acute lesions to predominant hyperautofluorescence in the healed stage (Figure 6). The FAF signals were regarded as a strong indicator to the status/health of RPE cells.

In another prospective consecutive case series of twelve patients with SC or SLC, all underwent serial FAF imaging [74]. Hypoautofluorescent halos surrounding the edges of hyperautofluorescent lesions were seen and correlated with active inflammation as assessed by FA.

Transitional SC is an intermediate stage between active and inactive inflammation. FA indicates that most or all of the inflammation has subsided. FAF images show a hypoautofluorescent line that surrounds all edges of the hyperautofluorescent lesions indicating that the SC lesions are stable and subsequently they do not increase in size. Inactive lesions are characterised by FAF images that are dark with very sharp borders due to complete loss of fluorophores. There is no hyperautofluorescence at the edge and this pattern correlates with inactive inflammation in FA and lesions that are clinically stable and in remission. These findings led to a paper in which SLC was differentiated from SC based on the FAF image findings. In this study FAF images of SLC lesions demonstrated a variegated pattern of hypo- and hyperautofluorescence signals that were distinct from the homogenous, contiguous hypoautofluorescence typically seen in SC [22, 75].

In these situations, FAF has proved to be a useful and easy to use clinical tool that can be employed to evaluate the extent of the affected area. FAF highlights subtle activity within the lesions, which can otherwise be easily missed. It is suggested that it can be used with caution to differentiate SC from SLC; however further studies are warranted. FA continues to be the gold standard imaging technique in cases where CNV is suspected and OCT remains very useful for monitoring disease activity.

### 3.2. Other Infectious Conditions

The use of FAF in the management of infectious uveitis has only been sporadically evaluated, with few reports regarding fluorescence patterns in different conditions. In patients with ocular syphilis, a hyperautofluorescence pattern, overlying the retinal lesion, has been described. As systemic antibiotic treatment is initiated this pattern resolves with a return to a normal autofluorescence pattern upon disease remission [76]. In a single case report of an immunocompromised patient who developed progressive outer retinal necrosis, secondary to varicella zoster virus, a stippled hyperautofluorescence pattern within extensive zones of hypoautofluorescence was noted, which corresponded to widespread RPE and outer retinal damage [77]. In patients with active cytomegalovirus retinitis a hyperautofluorescent area, corresponding to the advancing border of active retinitis, has been observed. However, later scans revealed a varied pattern of FAF, limiting its usefulness in monitoring disease progression and resolution [78].

## 4. Conclusion

Generally, in posterior uveitides, hyperautofluorescence indicates disease activity while quiescent disease and areas of chorioretinal atrophy or scarring are hypoautofluorescent. Fundus autofluorescence has recently been recognized as a useful noninvasive modality that is accurate in detecting early disease activity and extent of RPE damage. It serves in understanding pathophysiologic mechanisms and proves to be a valuable prognostic indicator in many posterior uveitides. Interestingly, in some conditions such as PIC, SLC, PVRL, MEWDS, and BSCR, FAF imaging reveals more widespread areas of disease activity than can be seen clinically. Autofluorescence is an adjunctive and helpful noninvasive tool in conjunction with other imaging modalities such as OCT, FFA, and ICG.
